# Collaborative Processing of Wearable and Ambient Sensor System for Blood Pressure Monitoring

**DOI:** 10.3390/s110706760

**Published:** 2011-06-28

**Authors:** Masayuki Nakamura, Jiro Nakamura, Guillaume Lopez, Masaki Shuzo, Ichiro Yamada

**Affiliations:** 1 NTT Energy and Environment Systems Laboratories, Nippon Telegraph and Telephone Corporation, Morinosato Wakamiya, Atsugi-shi, Kanagawa Pref. 243-0198, Japan; E-Mail: nakamura.jiro@lab.ntt.co.jp; 2 School of Engineering, The University of Tokyo, 7-3-1 Hongo, Bunkyo-ku, Tokyo 113-8656, Japan; E-Mails: guillaume@lelab.t.u-tokyo.ac.jp (G.L.); shuzo@lelab.t.u-tokyo.ac.jp (M.S.); yamada@mech.t.u-tokyo.ac.jp (I.Y.)

**Keywords:** blood pressure sensor, wearable sensor, ambient sensor, synchronization

## Abstract

This paper describes wireless wearable and ambient sensors that cooperate to monitor a person’s vital signs such as heart rate and blood pressure during daily activities. Each wearable sensor is attached on different parts of the body. The wearable sensors require a high sampling rate and time synchronization to provide a precise analysis of the received signals. The trigger signal for synchronization is provided by the ambient sensors, which detect the user’s presence. The Bluetooth and IEEE 802.15.4 wireless technologies are used for real-time sensing and time synchronization. Thus, this wearable health-monitoring sensor response is closely related to the context in which it is being used. Experimental results indicate that the system simultaneously provides information about the user’s location and vital signs, and the synchronized wearable sensors successfully measures vital signs with a 1 ms resolution.

## 1. Introduction

Wearable sensor technology is promising in relation to continuous health monitoring [1,2]. Some wearable biosensors have been developed such as heart rate sensors [3–7], electrocardiogram (ECG) sensors [8–18], and blood pressure sensors [19–22]. In particular, blood pressure sensors play an important role in detecting hypertension and cardiovascular diseases. However, conventional blood pressure sensors employ a cuff, which is obtrusive and restricts the user’s movement. Cuff-less blood pressure sensors are preferred for continuous monitoring. One such sensor functions by calculating the pulse transit time (PTT) using a photoplethysmography (PPG) sensor and an ECG sensor [19]. The PTT has to be measured with a millisecond order to estimate systolic blood pressure. These two sensors are attached to different parts of the body, thus making the sensor wires cumbersome during measurements. Wireless body sensor network technology is needed for such wearable sensing. Collaboration between the wearable sensors, and in particular time synchronization, is needed if we are to measure the PTT with a millisecond order and a high sampling rate.

Context awareness is also important in health monitoring applications [23,24]. Vital signs depend on their context, which can include information about physical activity, time and place. Ambient sensors that acquire context information can also improve the activity recognition performance of wearable acceleration sensors [25]. Taking context awareness into consideration, we can focus on particular features of vital signs during continuous health monitoring. The collaborative processing of wearable and ambient sensors provides useful information about our daily activities.

In this paper, first we describe our collaborative wearable and ambient sensors. Especially, we emphasize the precise synchronization of the multiple wireless wearable sensors and the collaboration between the wearable sensors and the ambient sensors. Our system consists of sensor networks which enable synchronization, real-time sensing and context-aware wearable sensing. Then we report PTT measurement experiments that we conducted using PPG and ECG wearable sensors and ambient occupancy sensors. We also show that how the systolic blood pressure depends on the PTT using a conventional blood pressure sensor. Finally we discuss the experimental results and evaluate the proposed system.

## 2. Collaborative Processing of Wearable and Ambient Sensors

It is essential to synchronize the clocks of wirelessly distributed wearable sensors for the simultaneous sensing of ECG and PPG because the PTT must be measured with a 1 ms resolution. The sensor readings from different sensors should be provided with time stamps especially when there are delays in data transmission [26]. In addition, the clocks exhibit frequency drift and so their frequencies are not identical. The differences range from several to tens of ppm and can cause a time lag and an error in the PTT measurements. These facts show that the wearable sensors must collaborate; in other words, time synchronization is necessary for precise data acquisition.

In addition to the collaboration between the wearable sensors, ambient sensors play important roles in context-aware wearable sensing. The context is important information for what concerns health monitoring. There are many situations in which we want to focus on the details of a user’s vital signs. These include when the user has just woken up, when the user is walking up stairs, and when the user is working hard at home. The system synchronizes the clocks and begins to operate when a particular event occurs. This selective sensing also enables the system to operate with low power consumption. Simultaneous wearable and ambient sensing allows us to realize context aware wearable sensing.

Sensor fusion is a signal processing technology that improves the sensing ability using multiple sensors [27]. The collaboration between wearable and ambient sensors in this paper represents a kind of sensor fusion technology. However, we concentrate on its network aspect. It is not only a multisensor fusion system but also a networked and synchronized sensor system. The wearable sensors are activated or synchronized when they receive a signal directly from the ambient sensor that fired. Then the wearable sensors transmit the sensor readings and the time stamp to a PC.

**Figure 1.**
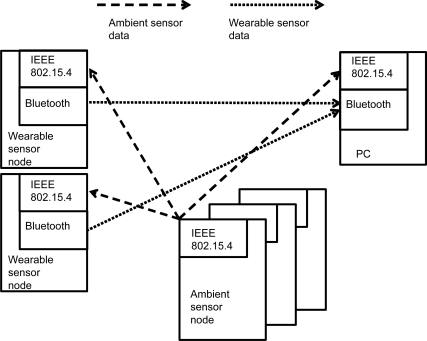
Wearable and ambient sensors.

Figure 1 shows the proposed wearable and ambient sensor system. It comprises wireless ambient sensors, wireless wearable sensors and a PC that collects the sensor data. For the ambient sensors such as occupancy sensors, an IEEE 802.15.4 radio was used to transmit the data. The ambient sensors provide intermittent data depending on the user’s presence.

For wearable sensors such as ECG and PPG, the data transmission must be fast because the wearable sensor sampling rate is as high as 1 kHz. The wearable sensor node uses a Bluetooth module to transmit the sensor data. In addition, the wearable sensors are also equipped with an IEEE 802.15.4 radio to receive the ambient sensor data. The detection of the user’s presence is a trigger event that activates the two wearable sensors at designated points. Thus, the wearable sensor responses are closely related to the ambient sensor responses. The PC can acquire the occupancy sensor data and synchronized real-time ECG and PPG streaming. Although the IEEE 802.15.4 and the Bluetooth have the same frequency (2.4 GHz), there is little interference between them due to Bluetooth’s frequency hopping spread spectrum (FHSS) approach and the direct sequence spread spectrum (DSSS) mechanism of the IEEE 802.15.4.

## 3. Experimental Setup

**Figure 2.**
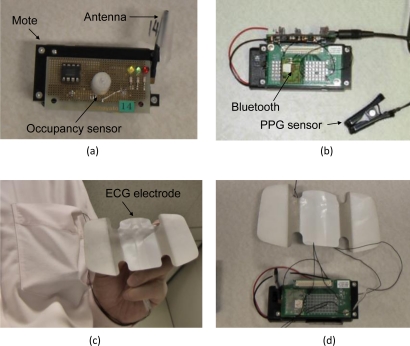
Sensor nodes. (**a**) Occupancy sensor; (**b**) PPG sensor; (**c**) and (**d**) ECG sensor.

**Figure 3.**
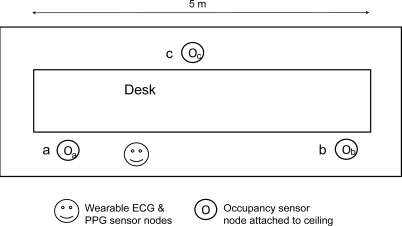
Sensor deployment.

We used Crossbow motes, MICAzs (radio: IEEE 802.15.4) with a 10-bit A/D converter for wireless sensor networks (Figure 2). The wearable sensor nodes were also equipped with Bluetooth modules (ZEAL-C01, ADC Technology Inc.) to transmit sensor data. The Bluetooth module was connected to the UART port of the mote. We used an Ag/AgCl electrode (Vitrode T, Nihon Kohden Corp.) as an ECG sensor. The signal was filtered with a low-pass filter (cut-off frequency: 100 Hz). We used a transmission photosensor (ear sensor, Combi Wellness Corp.) as a PPG sensor. The PPG sensor was attached to the left earlobe. The other motes were equipped with occupancy sensors (AMN13112, Panasonic Electric Works) as ambient sensors. These sensors were connected to the A/D converter ports of the motes. For the wearable and ambient sensor measurement experiment, the occupancy nodes were attached to the ceiling (3 m high) to detect the user’s presence (Figure 3). They transmit a signal using the IEEE 802.15.4 radio when they detect the presence of the user. When the wearable sensors receive the detection signal, they simultaneously begin to perform measurements with a sampling rate of 1 kHz and transmit the data to the PC using Bluetooth for 15 s. Average heart rate and PTT during the period are calculated.

**Figure 4.**
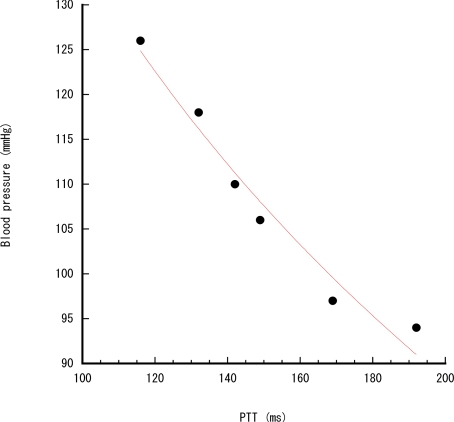
Blood pressure *vs.* PTT.

First we measured the synchronization error between the two wearable sensor nodes using a logic analyzer (LAP-16128U, SkaTec GmbH) when an occupancy sensor node fired. Then, we conducted a PTT measurement experiment in a workplace. The user with the wearable sensors moved from point a to point c through point b, and stood still for 20 s at each point. The PTT measurement was assumed to be required only at each point. When the user reached each point, the occupancy sensor fired and the wearable sensors began to operate. The PC collected the time stamp and sensor readings from the wearable sensors using a Bluetooth receiver (PTM-UBT5, Princeton Technology Ltd.) and the occupancy sensor data using an IEEE 802.15.4 radio receiver (mote base station). Finally, we measured the PTT and systolic blood pressure simultaneously according the protocol described in [19], in order to calibrate the cuff-less systolic blood pressure calculation from PTT. The user wore the PPG sensor, the ECG sensor and a conventional blood pressure measurement device with a cuff (HEM-7080IC, Omron Corp). He exercised and rested for the blood pressure measurement. The systolic blood pressure is estimated by a logarithmic function [19], which calibration’s result from PTT is shown on Figure 4.

## 4. Results

We conducted twenty measurements of the synchronization error between the two wearable sensor nodes. The resultant average synchronization error was 0.04 ms. The value was smaller than the required resolution of PTT, *i.e*., 1 ms. This result indicates that the time synchronization was successful and the wearable sensors simultaneously measured each signal and could provide sensor signals with a 1 ms resolution.

**Figure 5.**
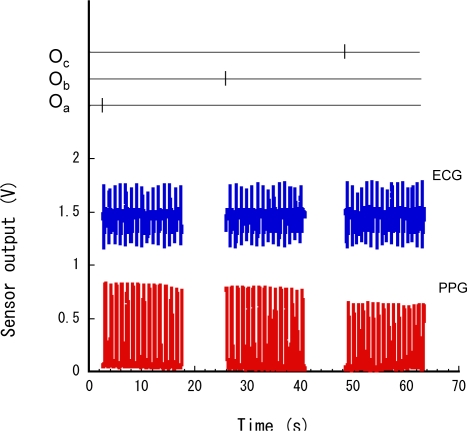
Sensor responses.

Figure 5 shows typical responses of the three occupancy sensors, the ECG sensor, and the PPG sensor in the wearable and ambient sensor measurement experiment. This result indicates that each sensor was synchronized at each point. The occupancy sensors detected the user at each point, and the ECG and PPG sensors were activated synchronously by the occupancy sensors. The wearable sensors did not operate except at the three points. The firing of the occupancy sensors denotes the user’s locations. Thus, this system can acquire the user’s vital signs in relation to the locations.

**Figure 6.**
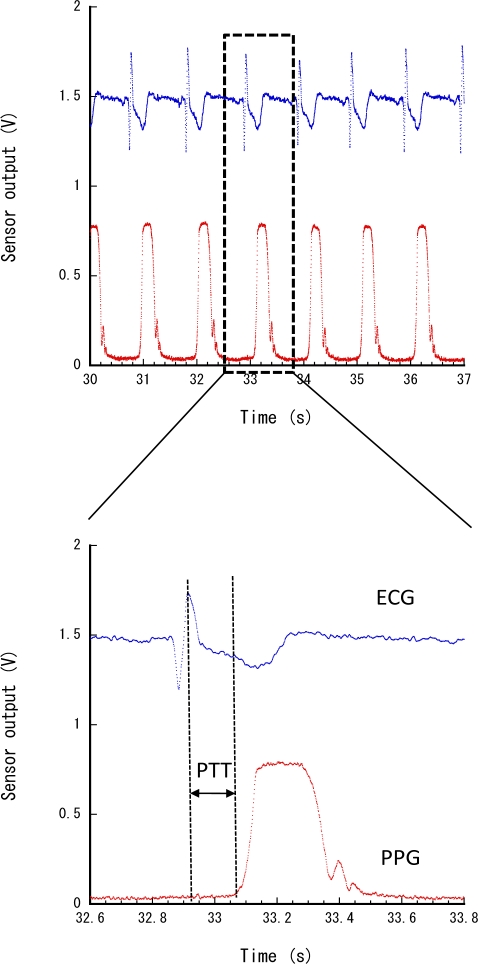
PTT estimated from ECG and PPG.

Table 1 shows the estimated average heart rate, PTT and blood pressure at each point. The heart rate was calculated from the intervals between the peaks of the ECG signal, that is, *h* = 60/*r*, where *h* is the heart rate and *r* is the interval between ECG peaks. The PTT was measured as the time between the ECG signal’s peak and the PPG signal’s foot (Figure 6), and blood pressure calculated according the function previously described. According to these results, the user’s physiological status appears stable at each point because he walked only for a short time and then rested in this experiment.

## 5. Discussions

The Bluetooth data rate is greater than that of the IEEE 802.15.4. We used Bluetooth to transmit the wearable sensor data at a 1 kHz sampling rate so that we successfully acquired real-time ECG and PPG streaming. At the same time, the wearable sensors were equipped with the IEEE 802.15.4 radio for time synchronization. The two radios enable the wearable sensors to perform real-time sensing and collaborate with the ambient sensors. If real-time ECG and PPG streaming is not required, the wearable sensors can store the data in their onboard flash memories and later transmit these data using the IEEE 802.15.4 [28]. In this case, the wearable sensors do not require Bluetooth modules. The Bluetooth consumes more power than the IEEE 802.15.4 radio. To reduce power consumption, we can utilize only the IEEE 802.15.4 radio for the sensor network.

The wearable and ambient sensors collaborated successfully in this experiment. We obtained the ECG, PPG, heart rate, PTT and blood pressure associated with the user’s location. Ambient sensors include cameras, switches, and light sensors, which can provide the context. These sensors characterize a particular event, action and location. Although continuous health monitoring is important, we often want to focus on the health status at a particular point. The collaboration between wearable and ambient sensors enables the proposed system to satisfy this demand.

**Figure 7.**
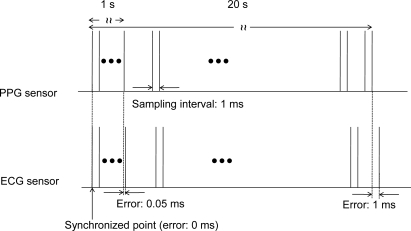
Accumulated synchronization error.

The synchronization error of the wearable system was 0.04 ms, which does not affect the PTT measurement with a 1 ms resolution. Instead, the frequency drift and difference between the clocks of the wearable sensors affect the measurement (Figure 7). If the measured value of the frequency drift and difference is 50 ppm, the resultant accumulated synchronization error is calculated as 50 μs in a 1 s measurement (50 × 10^−6^ s). This leads to an accumulated synchronization error of 1 ms for a 20 s measurement (50 × 10^−6^ × 20 = 10^−3^ s). A PTT measurement requires a 1 ms resolution. Therefore, additional time synchronization is needed for measurements longer than 20 s. Alternatively, it is possible to realize a longer measurement without frequent synchronization by calibrating the frequency difference. The smaller the frequency difference becomes, the smaller the accumulated synchronization error becomes. In this paper, the measurement time was limited to 15 s to avoid the possibility of an accumulated synchronization error larger than 1 ms.

The PTT can be converted to blood pressure to provide a more general index [29]. In this experiment, we chose a simple and physically undemanding movement pattern for the user, and the wearable sensors were operated during rest periods, so their responses were stable. Though our aim was to test the basic performance of the collaborative system, if the user exercised during the experiment, the blood pressure would vary and we would obtain sensor responses corresponding to the extent of the exercise [19].

User movement such as running induces noise in the ECG and PPG responses. We measured the ECG and PPG at rest to avoid noise in this experiment. Continuous health monitoring is required even during walking and running. One approach to noise cancellation involves using acceleration sensors. The acceleration sensors monitor the movement, and the noise induced by the movement is assumed to be cancelled by signal processing techniques of the acceleration sensors’ responses such as adaptive filters [30]. This remains as future work.

## 6. Conclusion

We described collaborative wearable wireless and ambient sensors that acquire vital signs such as blood pressure in context during daily activities. The system enabled us to realize real-time and synchronized ECG and PPG streaming and to measure the heart rate and the PTT with a 1 ms resolution. We evaluated the synchronization errors and confirmed that they were small enough to allow us to synchronize the wearable sensors. We also showed the wearable sensors functioned as a blood pressure sensor. In addition to the collaboration between the wearable sensors, we described collaboration between wearable and ambient sensors. Context awareness is also an important factor in health monitoring. In this paper, we associated vital signs with user locations. We performed an experiment using the wearable and ambient sensors and confirmed that they operated collaboratively. The wearable sensors can operate selectively with low power consumption. This selective sensing enables us to focus on the vital signs at a particular point. We believe this approach will be useful for wearable health monitoring.

## Figures and Tables

**Table 1.** Heart rate, PTT and blood pressure estimated from ECG and PPG.

	**Point a**	**Point b**	**Point c**
Average heart rate (bpm)	58	59	63
Average PTT (ms)	159	165	162
Average blood pressure (mmHg)	104	101	102
